# Clustering of unhealthy food around German schools and its influence on dietary behavior in school children: a pilot study

**DOI:** 10.1186/1479-5868-10-65

**Published:** 2013-05-24

**Authors:** Christoph Buck, Claudia Börnhorst, Hermann Pohlabeln, Inge Huybrechts, Valeria Pala, Lucia Reisch, Iris Pigeot

**Affiliations:** 1 Leibniz Institute for Prevention Research and Epidemiology - BIPS, Bremen, Germany; 2 Department of Public Health, Ghent University, Ghent, Belgium; 3 International Agency for Research on Cancer, Dietary Exposure assessment group, Lyon, France; 4 Department of Preventive and Predictive Medicine, Nutritional Epidemiology Unit, Fondazione IRCSS Istituto Nazionale dei Tumori, Milan, Italy; 5 Department of Intercultural Communication and Management, Copenhagen Business School, Copenhagen, Denmark

## Abstract

**Background:**

The availability of fast foods, sweets, and other snacks in the living environment of children is assumed to contribute to an obesogenic environment. In particular, it is hypothesized that food retailers are spatially clustered around schools and that a higher availability of unhealthy foods leads to its higher consumption in children. Studies that support these relationships have primarily been conducted in the U.S. or Australia, but rarely in European communities. We used data of FFQ and 24-HDR of the IDEFICS study, as well as geographical data from one German study region to investigate (1) the clustering of food outlets around schools and (2) the influence of junk food availability on the food intake in school children.

**Methods:**

We geocoded food outlets offering junk food (e.g. supermarkets, kiosks, and fast food restaurants). Spatial cluster analysis of food retailers around child-serving institutions was conducted using an inhomogeneous K-function to calculate global 95% confidence envelopes. Furthermore, a food retail index was implemented considering the kernel density of junk food supplies per service area, adjusted for residential density. We linked the food retail index to FFQ and 24-HDR data of 384 6- to 9-year-old school children in the study region and investigated the impact of the index on food intake, using multilevel regression models adjusted for sex, age, BMI, parent’s education and income, as well as adjusting for over- and underreporting of food intake.

**Results:**

Comparing the 95% confidence envelopes to the observed K-function, we showed that food stores and fast food restaurants do not significantly cluster around schools. Apart from this result, the food retail index showed no effect on BMI (*β*=0.01,*p*=0.11) or food intake variables assessed by FFQ and 24-HDR.

**Conclusion:**

In the built environment of the German study region, clustering of food retailers does not depend on the location of schools. Additionally, the results suggest that the consumption of junk food in young children is not influenced by spatial availability of unhealthy food. However, investigations should be replicated in other European communities to increase environmental variability.

## Background

Urban availability of fast food stores and restaurants in the living environment of children is assumed to contribute to an obesogenic environment. In particular, it is hypothesized that food outlets and fast food restaurants are clustered in school-neighborhoods and that a higher spatial availability of unhealthy food results in increased consumption in children. However, previous findings strongly differ with regard to study design, main outcome variables, food store definitions, and measures of food availability [1-3]. For example, Powell et al. [4] found a positive association between the availability of convenience stores and body mass index (BMI) in a large sample of U.S. adolescents. Furthermore, a study based on ninth grade students from 879 public schools in California showed that the presence of a convenience store within a 10-minute walking distance of a school was associated with a higher rate of overweight students compared to schools without nearby convenience stores, but nearby fast food restaurants and supermarkets were not associated with the prevalence of overweight in schools [5].

However, another national study of U.S. adolescents showed that fast food availability was not associated with weekly frequency of fast food consumption in non-urban and low- or high-density urban areas [6]. Investigating youths in 179 Canadian schools, Seliske et al. showed that the exposure to various types of food retailers in school neighborhoods was not associated with an increased likelihood of being overweight [7]. For elementary school children in the U.S., Sturm and Datar also found no effects of food outlet density on changes in BMI at the neighborhood level [8].

The great heterogeneity in study designs, measures etc. limits a comparison of findings regarding the community food environment [9-11]. Furthermore, these findings can hardly be transferred to European communities, because structures of the studied communities differ in many aspects from European communities [3]. For instance, communities in the U.S. and Europe differ with regard to urban patterns, location of stores, and range of products, as well as travel behavior and eating behavior of consumers [12]. Portion size of fast food restaurants also strongly differs between the U.S. and Europe [13].

Because the community food environment in Europe is an understudied area [3,9,11], we conducted a pilot study of the food environment in one German study region of the IDEFICS study (Identification and prevention of dietary- and lifestyle-induced health effects in children and infants) [14]. Our main objective was to adopt two methods: the K-function that was used to investigate clustering of food supply around schools and the density approach to assess the spatial availability of food supply at the community level.

Using the K-function, Austin et al. found that fast food restaurants in Chicago were significantly clustered in areas within a short walking distance from schools with about 3 to 4 times as many fast food restaurants within 1.5 km from schools than would be expected if the restaurants were randomly distributed throughout the city [15]. Another study in New Zealand also found a significant spatial clustering of fast food outlets within a 1.5 km radius around schools [16]. In these studies, the K-function [17,18] compared the observed clustering of fast food restaurants to the expected clustering under the assumption of complete spatial randomness (CSR). However, the probability of the location of food stores is not the same throughout an entire study area. Thus, an inhomogeneous K-function is more appropriate for urban analyses [19,20].

Density of food stores was measured differently to assess the availability of or the accessibility to food stores or fast food restaurants [9,10]. Commonly used measures are, for example, simple density approaches, i.e. number per area [21,22] or per capita [7], or kernel density approaches that give a weighting to stores or restaurants depending on the distance to the point of observation [23].

Using these methods, we first analyzed the spatial distribution of food stores around primary schools to investigate whether junk food choices are clustered around child-serving institutions. Second, we calculated food supply using the kernel density of food stores adjusted for residential density to eventually compare environmental food supply to dietary data assessed by Food Frequency Questionnaires (FFQ) and 24-hour dietary recalls (24-HDR) in the German study region of the IDEFICS study.

## Methods

### The IDEFICS study

Geographical data were collected in one German study region of the IDEFICS study which is an Integrated Project within the Sixth Framework Programme of the European Commission. The IDEFICS study used a multicenter survey design of a population-based cohort to investigate the etiology of selected diet- and lifestyle-related diseases. More than 16,000 2- to 9-year-old children from eight European countries were included in the baseline survey (T0) that was conducted from October 2007 until May 2008. During the baseline survey, dietary habits, social, environmental, and family factors, physical activity, and anthropometric indices were assessed [14]. Moreover, the study developed, implemented, and evaluated strategies for primary prevention of overweight and obesity in this age group. In Germany, ethical approval for the survey was given by the ethical committee of the University of Bremen [14].

### Environmental analysis

#### Geographical data

This study was based on one study region of the IDEFICS study which is Delmenhorst, Lower Saxony, Germany, with an area of 62.36 km^2^ and about 77,300 residents^a^.

The street addresses of food stores and restaurants were collected in 2008 using public domain data sources^b^. A total of 183 stores and restaurants were listed and validated by field observations in 2008, as suggested in Wang et al. [24]. As a consequence, 50 addresses had to be removed and 55 new stores and restaurants were added to the list. Finally, the remaining 188 stores and restaurants including all opportunities for unhealthy food (i.e., fast food restaurants, snack bars, kebab shops, bakeries, kiosks, small grocery stores, and chain supermarkets) were digitalized in ArcGIS10.

In most publications, mainly fast food restaurants were considered to assess the environmental availability of unhealthy foods whereas supermarkets and grocery stores were considered as proxies for healthy food [1,4,25]. However, supermarkets, grocery stores, and other types of stores offer a wide range of unhealthy foods. Thus, in addition to fast food restaurants, all stores offering unhealthy food choices such as supermarkets, grocery stores, bakeries, and kiosks were taken into account in our analyses. To ensure that this classification of unhealthy food retailers does not affect the results compared to classifications in the literature, we conducted sensitivity analyses considering the 49 fast food restaurants and convenience stores on the one hand and only the 25 supermarkets on the other hand.

**Figure 1:**
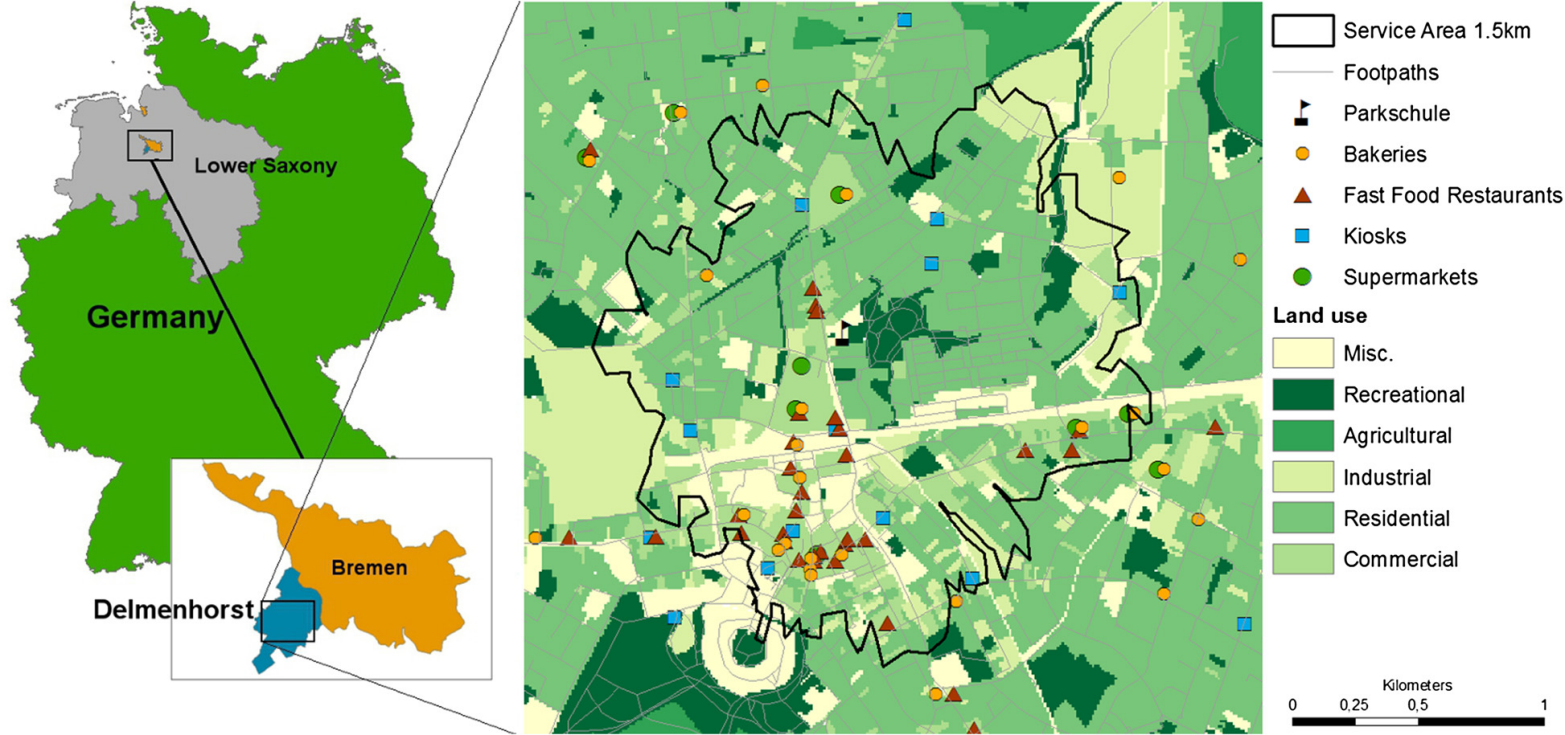
**Study area.** Example of a 1.5 km service area and food supply around one school in the study area Delmenhorst, Germany.

As a reference map, the AK5 (official map, scale 1:5000) was obtained from the land registry office of Lower Saxony. The map contains information such as parcels and parcel identification numbers, different classes of buildings, land use types, and street names. Land use records were used to identify commercial land use as potential locations for food stores. In addition, we obtained boundaries of districts and subdistricts, including the number of residents, and a list of primary schools from the municipality of Delmenhorst. The municipal geospatial information system (Kommunales Raumbezogenes Informationssystem (KRIS)) of Delmenhorst provided a complete dataset of sidewalks that we used to implement network-based service areas of 1.5 km around each school using the *network analyst*-tool in ArcGIS 10 (see Figure 1). The distance of 1.5 km was chosen in accordance with Austin et al. [15]. A larger area would have reduced environmental variability of the food retail index, whereas a smaller area would have reduced the sample size (see study data for details). Moreover, 80% of the school children walk/cycle to and from school if they live within 1.5 km or less from school.

#### Analysis of spatial clustering

Ripley’s bivariate K-function analyzes the spatial correlation of point locations (e.g., fast food restaurants) around locations of interest (e.g., schools) in order to detect potential clusters depending on the distance between these locations [17]. In two studies of the community food environment, the K-function was used to investigate clustering of fast food restaurants and convenience stores around schools [15,16]. In general, this method is applied to compare an empirical K-function with the expected K-function. Here, the empirical K-function is calculated as the observed number of restaurants and stores around schools depending on their distance to the considered schools. Based on the assumption of complete spatial randomness (CSR), point patterns are simulated to obtain 95% confidence limits of the expected K-function and to test the statistical significance of the observed clustering [18]. For the homogeneous K-function, point patterns are simulated using a stationary probability surface which means that each potential location of a food store has the same probability in the whole study area. However, food stores cluster within the urban environment and the probability of the location of food stores can vary as one can expect a higher probability in the center of a city or near major streets which leads to the concept of an inhomogeneous K-function. This accounts for the non-stationary distribution of food stores in the urban environment which may therefore improve the detection of clustering [19,20].

To compare the performance of these approaches, we used both the homogeneous bivariate K-function [17]

(1) $${\hat{K}}_{\text{ij}}(r)=\frac{\left|W\right|}{n\cdot m}\overset{n}{\underset{i=1}{\sum }}\overset{m}{\underset{j=1}{\sum }}w_{s_{i}^{f},\;s_{j}^{s}}I(|s_{i}^{f}-s_{j}^{s}|<r)$$

and the inhomogeneous bivariate K-function [20]

(2) $${\hat{K}}_{\text{ij}}^{\text{inhom}}(r)=\frac{1}{\left|W\right|}\overset{n}{\underset{i=1}{\sum }}\overset{m}{\underset{j=1}{\sum }}w_{s_{i}^{f},\;s_{j}^{s}}\frac{I\;(|s_{i}^{f}-s_{j}^{s}|<r)}{{\hat{\lambda }}_{i}(s_{i}^{\text{cl}}){\hat{\lambda }}_{j}(s_{j}^{s})},$$

to identify clusters of food retailers $s_{i}^{f},i=1,\ldots ,n$ (n=188 in this study), around primary schools $s_{j}^{s},j=1,\ldots ,m$ (m=14), for any distance r∈R in the study area $W\subset R^{2}$, where |*W*| is the size of the study area and $w_{s_{i}^{f},s_{j}^{s}}$ is the ‘reduced sample’ estimator as described by Ripley [17].

The K-function as defined in equation (2) accounts for the inhomogeneous clustering of food stores within the study area using a non-parametric ‘leave-one-out’ kernel estimate ${\hat{\lambda }}_{j}(s_{j}^{s})$ for the point pattern $s_{j}^{s}$ of schools. To estimate the probability surface of food stores, we calculated the kernel estimate ${\hat{\lambda }}_{i}(s_{i}^{\text{cl}})$ for a point pattern $s_{i}^{\text{cl}}$ which was implemented based on commercial land use types and describes potential locations of food stores and restaurants [20]. The empirical K-function ${\hat{K}}_{\text{ij}}^{\text{inhom}}$ is compared to the expected K-function which is $K_{\text{ij}}=K_{\text{ij}}^{\text{inhom}}(r)=\pi r^{2}$ for the homogeneous and the inhomogeneous case under the assumption that the point patterns $s_{i}^{f}$ and $s_{j}^{s}$ are spatially independent. Thus, the inhomogeneous K-function investigates the null hypothesis that the point patterns $s_{i}^{f}$ and $s_{j}^{s}$ are realizations of different probability surfaces, here ${\hat{\lambda }}_{i}(s_{i}^{\text{cl}})$ and ${\hat{\lambda }}_{j}(s_{j}^{s}),$ respectively.

We used the kcross and the kcross.inhom function of the spatstat package in R^c^ to compare the empirical K-functions (1) and (2) to the corresponding expected K-function. In addition, we calculated global 95% confidence envelopes with the envelope function for both K-functions. With regard to (1), the confidence envelopes are calculated based on simulated random point patterns. For the inhomogeneous case (2), the confidence envelopes are calculated based on kernel estimates of $s_{i}^{\text{cl}}$ and $s_{j}^{s}$ that were calculated by the density.ppp function [17,18,20].

#### Food retail index

**Figure 2:**
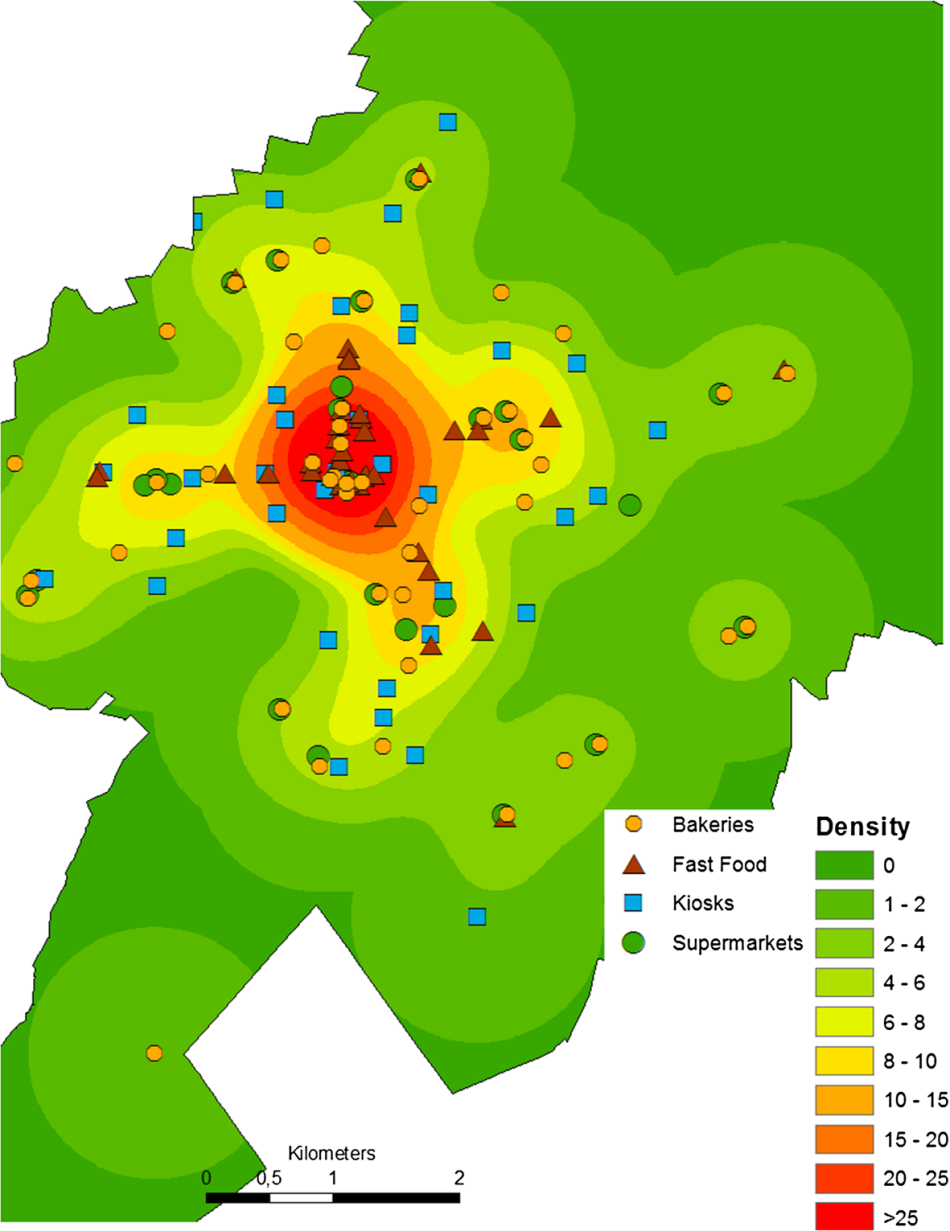
**Kernel density approach.** Kernel density of food retailer, i.e. number per km^2^, in the study area Delmenhorst.

As suggested by Moore et al. [23], the spatial availability of food retailers was assessed by a kernel density approach. In general, the kernel density estimates the number of stores and restaurants per area which was divided by the number of residents per area to obtain a standardized variable describing the food supply around schools. In detail, we used the kernel density

(3) $$\hat{\lambda }(s_{l}^{f})=\frac{1}{h^{2}}\overset{n}{\underset{i=1}{\sum }}\;K\;\left(\frac{\left|s_{l}^{s}-s_{i}^{f}\right|}{h}\right),$$

which is the inhomogeneous estimate of the mean number of food stores and restaurants $s_{i}^{f}\in R^{2},i=1,\ldots ,n(n=18$), in the study area $W\subset R^{2}$. Each point is weighted by the kernel function K depending on the bandwidth *h*[26]. Here, the kernel density-tool in ArcGIS 10 uses a quadratic kernel function K, a bandwidth of h=1 km and a raster of 10 m*10 m cells $s_{l}^{c}\in W\subset R^{2}$, where the number *l*=1,…,*L* depends on the size of the study area *W*. The farther away a store or restaurant $s_{i}^{\text{food}}$ is located from a cell $s_{l}^{c}$, the lower the weight given by the function K. For a distance greater than the bandwidth, the weight is zero. For each cell, the weights of all stores within the bandwidth were summed up and standardized for square kilometers (see Equation (3)). Figure 2 shows the kernel density of supermarkets, food stores, and restaurants in the study region. The values of the kernel density represent the estimated number of food retailers per km^2^.

Finally, the food retail index was built on the ratio of the number of food retailers per resident that was proposed by Seliske et al. [7]. Therefore, the mean kernel density

(4) $${\bar{\Lambda }}_{A}=\frac{1}{L}\overset{L}{\underset{l=1}{\sum }}\hat{\lambda }(s_{l}),$$

of all cells $s_{l}^{c}\in A,l=1,\ldots ,L$, per service area $A\subset W\subset R^{2}$ was calculated and then divided by residential density *R*_*A*_, where the number of cells *L* depends on the size of the service area *A*. ${\bar{\Lambda }}_{A}$ estimates the mean number of food retailers per service area. The ratio ${\bar{\Lambda }}_{A}/R_{A}$ is then the number of food retailers per 1,000 residents, which we used as the food retail index. For sensitivity analyses, we calculated the food retail index using the mean kernel density (3) of fast food restaurants and convenience stores (n=49) per service area only, and of supermarkets (n=25) per service area only, which we called the fast food index and the supermarket index, respectively.

### Study data

In the German study region of IDEFICS, individual data of 610 6- to 9-year-old school children were available. Since we calculated the food retail indices using 1.5 km school service areas, we linked the environmental data to the individual data of 500 school children (82%) living up to 1.5 km away from their school.

In 465 children, dietary data were assessed using the computer-based 24-HDR “SACINA” (Self-Administered Children and Infants Nutrition Assessment) which was based on the validated HELENA-DIAT [27] that was originally developed for Flemish adolescents [28]. Proxies which were mainly the parents completed the 24-HDR under supervision of field staff and answered questions with regard to six meal occasions (breakfast, morning snack, lunch, afternoon snack, dinner, and evening snack).

Only 17.8% provided more than one 24-HDR record. To obtain an equal number of 24-HDR for each child, only the first recall day was included in the current analysis (including weekdays and weekend days). The uniquely coded food items were linked to country-specific food composition tables that were used to calculate overall energy intake (in kcal/day) as well as fat intake and carbohydrate intake (in g/day) for each child. Recalculated Goldberg cut-offs (age- and sex-specific) [29,30] were used to define underreporting, overreporting and plausible records [31]. Based on FFQ data, frequency of junk food consumption (sweetened drinks, chocolate or nutbased spreads, and three types of snacks, like crisps, chocolate bars, or candies) and simple sugar foods (fruit juices, sweetened drinks, sugar added cereals, sweetened milk, sweetened yoghurt, and four types of snacks, like chocolate bars, candies, cakes, or ice cream) were added up to frequencies per week and were also used as outcome variables.

Body weight was measured to the nearest 0.1 kg, and height was measured to the nearest 0.1 cm. BMI was calculated and converted to age- and sex-specific z-scores [32]. Weight status was defined based on the International Obesity Task Force BMI cut-offs [33]. Age and sex of the children, as well as the ISCED-level of the parents (International Standard Classification of Education) and net household income were considered as potential confounders in the statistical analyses. Due to missing values in the confounding variables, 81 cases had to be removed and the sample size decreased to 384 children providing complete information. Table 1 presents main characteristics of this study sample.

**Table 1:** Characteristics of the study sample of school children in the study region, Delmenhorst, Germany

**Characteristics**	**All**		**Boys**		**Girls**	
	**N**	(**%**)	**N**	(**%**)	**N**	(**%**)
Sample size	384	(100)	194	(50.5)	190	(49.5)
Weight status^*a*^						
Overweight	51	(13.3)	16	(8.3)	35	(18.4)
Obese	17	(4.4)	9	(4.6)	9	(4.2)
Income^*b*^						
Low	131	(34.1)	72	(37.1)	59	(31.1)
High	253	(65.9)	122	(62.9)	131	(69.0)
ISCED level^*c*^						
Low	134	(34.9)	68	(35.0)	66	(34.7)
High	250	(65.1)	126	(65.0)	124	(65.3)
	Mean ± SD		Mean ± SD		Mean ± SD	
Age	7.6 ± 0.7		7.6 ± 0.7		7.6 ± 0.8	
BMI z-score^*a*^	0.3 ± 1.2		0.2 ± 1.1		0.4 ± 1.3	

^*a*^:According to Cole et al. [33].

^*b*^:Net household income in categories, low: below €2,250.

^*c*^:Max. ISCED level of the parents, low: level 1 and 2 relates to lower secondary education and less.

### Statistical analyses

Descriptive statistics of the food retail indices as well as of the considered food intake variables were calculated stratified by weight status (normal vs. overweight/obese), household income, and educational status of the parents.

The influence of food supply per 1,000 residents on children’s food intake was investigated using two-level multivariate lognormal and normal regression models accounting for a cluster effect within school service areas (level one). Overall, six models were considered to analyze the influence of the food retail index on (1) frequency of fast food intake, and (2) simple sugar food intake (multivariate lognormal models), as well as the influence on (3) daily energy intake (kcal/day), (4) fat intake (g/day), (5) carbohydrate intake (g/day), and finally on (6) BMI z-scores of children (multivariate normal models). Sensitivity analyses were conducted using the fast food index as well as the supermarket index in all six models. All models were adjusted for age, sex of the child, ISCED level, and net household income, as well as over- or underreporting of food intake. Significance level was set to *α*=0.05 and statistical analyses were performed using SAS 9.2^d^.

## Results

**Figure 3:**
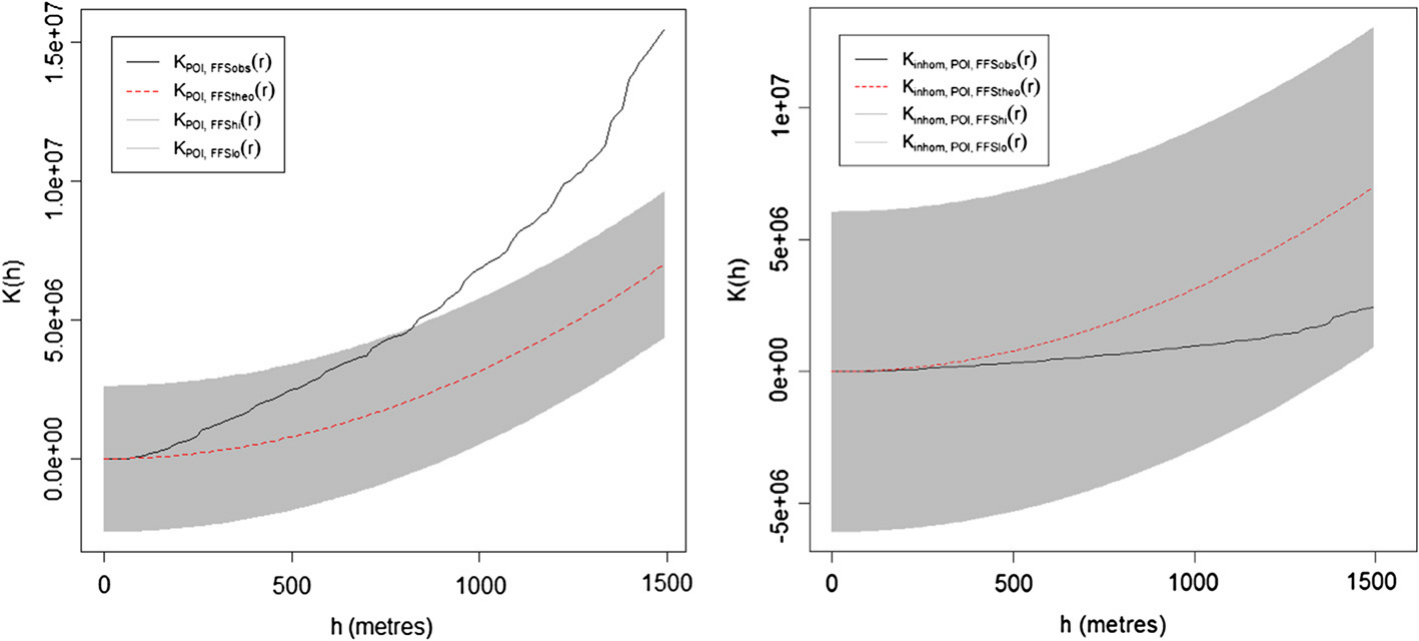
**Bivariate K-functions of food retailer around schools.** Empirical (black) and expected (red) bivariate K-functions (left: homogeneous, right: inhomogeneous) and 95% global upper and lower confidence limits (grey) showing the clustering of 188 food retailer around 14 schools depending on the distance from schools.

The empirical and expected K-functions with 95% global confidence limits for both the homogeneous and the inhomogeneous case are presented in Figure 3. The homogeneous K-function showed a significant clustering of food retailers around schools from a radius of about 750 m up to 1.5 km. Below this distance, food retailers did not significantly cluster around schools. The inhomogeneous K-function was also located within the 95% confidence limits and showed that food retailers are not clustered around schools for up to 1.5 km.

Descriptive statistics of the food retail index as well as of food intake variables showed some differences between groups of weight status or net household income and educational status (see Table 2). For overweight or obese children, mean and median values of the food retail index as well as both food frequency variables were higher than for normal weight children. For kcal, fat, and carb intake, higher mean and median values were found in normal weight children. For low household income or low ISCED levels, mean and median values were higher for the food retail index, for kcal and fat intake, and for weekly frequencies of junk food or simple sugar foods. Only mean carb intake in the high income category was higher than in the low income category.

**Table 2:** **Descriptive statistics (median, mean and standard deviation (M *± * SD) of the food retail index and of food intake variables stratified by weight status, household income and educational status**

		**Simple retail index**^***a***^		**Kcal / day**		**Fat (g)/day**		**Carb. (g)/day**		**Junk food**^***b***^		**Simple sugar foods**^***b***^	
**Class**	**N**	**Median**	**M ±SD**	**Median**	**M ±SD**	**Median**	**M ±SD**	**Median**	**M ±SD**	**Median**	**M ±SD**	**Median**	**M ±SD**
All	384	1.4	1.7 ±0.9	1,621	1,647 ±555	55.7	60.6 ±30.6	211	216.0 ±80.2	12	14.7 ±12.0	29	31.5 ±21.2
Weight status^*c*^													
Normal weight	316	1.1	1.7 ±0.9	1,637	1,675 ±557	56.9	62.0 ±31.4	214	220.0 ±80.9	12	14.5 ±11.7	30	31.4 ±20.6
Overweight/Obese	68	1.9	1.8 ±0.9	1,522	1,513 ±528	49.0	54.4 ±26.2	183	194.0 ±74.1	12	15.2 ±13.2	28	32.0 ±24.1
Household income^*d*^													
Low income	131	1.7	1.7 ±0.8	1,631	1,658 ±626	55.6	62.5 ±33.9	203	210.0 ±85.3	14	16.5 ±13.7	32	33.5 ±26.8
High income	253	1.1	1.7 ±0.9	1,621	1,641 ±515	55.2	59.7 ±28.8	211	219 ±77.5	12	13.7 ±10.8	28	30.5 ±17.5
Educational status^*e*^													
Low ISCED	134	1.7	1.8 ±0.9	1,636	1,637 ±612	56.7	60.6 ±30.9	215	219 ±77.9	14	17.7 ±15.2	32	33.0 ±25.3
High ISCED	250	1.1	1.6 ±0.9	1,621	1,652 ±522	54.7	60.7 ±30.6	200	210.0 ±84.4	11.5	13.0 ±9.4	28	30.7 ±18.7

^*a*^:Number of stores and restaurants per 1000 residents.

^*b*^:Frequencies per week.

^*c*^:According to Cole et al. [33].

^*d*^:Net household income in categories, low: below €2,250.

^*e*^:Max. ISCED level of the parents, low: level 1 and 2 relates to lower secondary education and less.

Table 3 presents results of the six regression models. In all models, the food retail index showed no effect on BMI, daily intake of kcal, fat, or carb, and on weekly frequencies of junk food or simple sugar foods. In Model 1 only high net household income and high educational status of the parents had a significantly negative effect on the BMI z-score of the children. Age was a significantly positive predictor of energy intake per day (Model 2) and girls had significantly lower energy intake per day and lower carb intake per day compared to boys (Model 2 and 4). Sensitivity analyses showed also no effects of the fast food index or the supermarket index on all six outcome variables, i.e. BMI z-score and food intake variables (results not shown). Particularly, the sensitivity analyses showed similar results for the confounding variables (age, sex, household income, and educational status) as the main analyses presented in Table 3.

**Table 3:** Results of normal and lognormal multilevel regression Models 1 - 6 investigating the effect of the food retail index on food intake and BMI adjusted for sex, age, household income and educational status as well as over- and underreporting (N = 384)

**Dependent** **variable**	**Model 1** **BMI z-score**^***a***^		**Model 2** **Energy (kcal/day)**		**Model 3** **Fat (g/day)**		**Model 4** **Carb. (g/day)**		**Model 5** **Junk Food**^***b***^		**Model 6** **Simple sugar foods**^***b***^	
	*β*	p-value	*β*	p-value	*β*	p-value	*β*	p-value	exp(*β*)	p-value	exp(*β*)	p-value
Food retail index^*c*^	0.11	0.17	-12.15	0.60	-2.08	0.24	2.43	0.65	1.04	0.57	0.99	0.87
Age	0.08	0.37	71.08	0.01	3.13	0.10	9.46	0.049	1.04	0.63	0.91	0.29
Sex (ref: male)	0.22	0.06	-127.4	0.001	-3.30	0.21	-19.9	0.003	0.98	0.86	0.95	0.68
High income (ref: low)^*d*^	-0.32	0.02	-21.22	0.62	-2.72	0.35	7.40	0.31	0.99	0.93	1.15	0.06
High ISCED (ref: low)^*e*^	-0.36	0.006	-28.75	0.50	-1.18	0.69	1.30	0.86	0.84	0.09	1.08	0.35

^*a*^:According to Cole et al. [33].

^*b*^:Frequencies per week.

^*c*^:Number of stores and restaurants per 1000 residents.

^*d*^:Net household income in categories, low: below €2,250.

^*e*^:Max. ISCED level of the parents, low: level 1 and 2 relates to lower secondary education and less.

## Discussion

Overall, the results of this pilot study of the community food environment did not support the hypothesis that environmental availability of foods contributes to unhealthy dietary patterns or higher rates of obesity in children in a German community.

First, the homogeneous K-function did not show any significant clustering of food retailers for up to 750 m around child-serving institutions. This finding is in contrast with the results of Day and Pearce [16] as well as Austin et al. [15] who found a significant clustering of fast food restaurants from about 100 m to 1.5 km distance from schools using a homogeneous K-function. This could be explained by differences in urban patterns between the U.S. and Germany. In Germany, schools generally have fewer students and are located across the entire city, while U.S. schools are commonly very large and are often located on transport nodal points of the street network which are also attractive for fast food restaurants. However, the result of the homogeneous K-function is misleading and does not imply that food retailers are clustered around schools from a distance greater than 750 m or that clusters of food retailers depend on the location of schools. In fact, food retailers are mainly located near major roads and in inner cities. Consequently, stores and restaurants cluster around the city center of the study area. The location of schools does not cluster in the study area, but most of the schools are within 1.5 km of the inner city. Thus, the ’natural’ cluster of food retailers in the inner city was detected by the homogeneous K-function at larger distances which was about 750 m in this study. Using the inhomogeneous K-function, simulated point patterns of food retailers were more likely to occur in urban patterns in which stores are genuinely located. Thus the inhomogeneous K-function showed no significant clustering of food retailers for the whole study area, since the inhomogeneous approach resulted in a lower slope in *K*(*h*) and produced wider confidence envelopes (see Figure 3) which improved the spatial cluster analysis considering the inhomogeneous simulation of point patterns [19].

Second, food availability around schools that was measured by the food retail index, did not show any significant effect on different food intake variables and individual BMI z-scores in our sample. Seliske et al. [7] who used the same ratio of food retailers per capita also did not find any association between the exposure to various types of food retailers and the likelihood of overweight in Canadian school-aged youth. Likewise, Sturm and Datar [8] found no effect of food outlet density on children’s BMI using number of food outlets per capita. Sensitivity analyses of our regression models considering only fast food restaurants and convenience stores as well as, and considering only supermarkets support these findings and strengthen the comparison to these studies [7,8,11]. Findings from other studies are difficult to compare since different measures were used to assess the community food environment and study regions in the U.S. and Australia strongly differ from European communities, particularly from our German study region [4,10,11,22,34].

Finally, several limitations of this pilot study have to be considered. Only parental proxy reports were available for the children’s dietary assessment. Thus, food intake of children that was not under parental control was not considered in the food intake variables and may especially include fast foods and sweets consumed at school. The study sample also might be too young, since 6- to 9-year-old children generally do not leave school grounds on their own, so that the chance of buying food is lower compared to adolescents. Only one 24-HDR was considered which does not reflect the usual intake due to the large day-to-day variation in diet and, in addition, social desirability could have influenced answers reported in the FFQ [35].

Table 2 showed the expected disparities in the food retail index between low and high net household incomes as well as low and high educational status with a higher junk food availability for low SES parents which is in line with findings from food environment studies in the U.S. [3], Canada [36], and parts of Europe [37], although the sample is slightly biased with regard to household income and educational levels with more parents belonging to higher SES groups. Furthermore, the well-known influences of household income and educational status on BMI could be replicated which may allow the conclusion that our study sample does not systematically differ from samples of larger studies.

Overall, findings from this study are based on a spatial analysis within one German community. Although this study did not provide evidence that spatial availability of unhealthy foods influences dietary behavior of children, replicating the methods presented in this article in larger studies using different European communities could increase environmental variability and could provide the opportunity to compare the influence of the spatial structure on dietary behavior, particularly between different communities. We therefore plan to perform a similar study in the second German study region of the IDEFICS study, as well as in the Italian and Swedish study regions.

## Conclusion

Environmental measures that were mainly used in U.S. studies to assess the community food environment provide a well-established toolkit which could be adopted to examine the impact of the food environment on food intake in German school children. However, both the spatial cluster analysis and the regression analyses including the food retail index did not support the hypotheses that unhealthy food supply is clustered around schools and that a higher level of availability of food retailers increases the intake of unhealthy foods leading to a higher proportion of obese children. Further studies should replicate the application of these methods in different European communities to increase environmental variability which would help to understand how the spatial structure of a community contributes to an obesogenic environment.

## Endnotes

^a^ Census data 2009: Facts and Figures from the City of Delmenhorst, Service of Urban Development and Statistics, 31.12.2009 (In German: “Zahlenspiegel 2009: Daten und Fakten aus der Stadt Delmenhorst, Fachdienst Stadtentwicklung und Statistik, Stand 31.12.2009”).

^b^http://www.gelbeseiten.de, http://www.delmenhorst.de, http://www.goyellow.de.

^c^http://www.r-project.org/.

^d^ PROC GLIMMIX; SAS version 9.2, SAS Institute Inc, Cary, NC.

## Competing interests

The authors declare that they have no competing interests.

## Authors’ contributions

IP and LR were responsible for the conceptualisation and the design of the environmental analyses within the IDEFICS study. CB, IH, and VP were responsible for the conceptualisation and implementation of the FFQ and 24-HDR data. HP conceptualized the statistical analyses. ChB conducted the environmental analyses and wrote the manuscript. All authors have contributed to the final manuscript and have approved it.
